# Molecular Identification of Selected *Streptomyces* Strains Isolated from Mexican Tropical Soils and their Anti-*Candida* Activity

**DOI:** 10.3390/ijerph16111913

**Published:** 2019-05-30

**Authors:** Diana Escalante-Réndiz, Susana de-la-Rosa-García, Raúl Tapia-Tussell, Jesús Martín, Fernando Reyes, Francisca Vicente, Marcela Gamboa-Angulo

**Affiliations:** 1 Unidad de Biotecnología, Centro de Investigación Científica de Yucatán (CICY), Calle 43 x 32 y 34, Chuburna de Hidalgo, Mérida, Yucatán 97205, Mexico; diana.escalante@cicy.mx; 2 Laboratorio de Microbiología Aplicada, División Académica de Ciencias Biológicas, Universidad Juárez Autónoma de Tabasco (UJAT), Villahermosa, Tabasco 86150, Mexico; susana.delarosa@ujat.mx; 3 Unidad de Energía Renovable, Centro de Investigación Científica de Yucatán (CICY), Calle 43 x 32 y 34, Chuburna de Hidalgo, Mérida, Yucatán 97205, Mexico; rtapia@cicy.mx; 4 Fundación MEDINA, Avda Conocimiento 34, 18016 Granada, Spain; jesus.martin@medinaandalucia.es (J.M.); fernando.reyes@medinaandalucia.es (F.R.); francisca.vicente@medinaandalucia.es (F.V.)

**Keywords:** *Streptomyces parvisporogenes*, antifungal, *Candida albicans*, carbazomycin G, PKS and NRPS genes

## Abstract

The increasing incidence of *Candida albicans* infections and resistance to current antifungal therapies has led to the search for new and more effective antifungal compounds. Actinobacterial species from the *Streptomyces* genus are recognized as some of the major producers of antimicrobial compounds. Therefore, the aims of this study were: (1) the identification of *Streptomyces* strains isolated from Mexican tropical acidic soils, (2) the evaluation of their antifungal activity on *C. albicans*, and (3) the exploration of the presence of polyketide synthase genes in their genome and antifungal secondary metabolites in their extracts. Four actinobacterial strains, isolated from previously unexplored soils with antibacterial antecedents, were selected. These strains were identified as *Streptomyces* *angustmyceticus* S6A-03, *Streptomyces manipurensis* S3A-05 and S3A-09, and *Streptomyces parvisporogenes* S2A-04, according to their molecular analyses. The ethanol extract of the lyophilized supernatant of *S. parvisporogenes* displayed the most interesting antifungal activity against *C. albicans,* with a minimum inhibitory concentration (MIC) of 0.5 mg/mL. Type I polyketide synthase (PKS-I) and non-ribosomal peptide synthase (NRPS) genes were detected in all strains. In addition, type II PKS genes (PKS-II) were also found in *S.* *manipurensis* S3A-05 and *S. parvisporogenes*. LC-UV-HRMS analysis of the active organic extract of *S. parvisporogenes* indicated the presence of the known antifungal compound carbazomycin G as the major component.

## 1. Introduction

*Candida albicans* is part of the normal microbiota of the mucosa from the oral cavity, gastrointestinal tract, and vagina in healthy humans. Under some circumstances and in susceptible individuals, it can cause superficial and systemic infections in immunocompromised situations due to their great adaptability to different host niches [1,2]. The recent increase in the number of *C. albicans* strains resistant to the antifungal drugs currently in use has triggered the search for new sources of antifungal molecules to effectively treat mycotic infections [3]. Unfortunately, antifungal therapies using the currently available drugs are limited by their toxicity, side effects, and the development of drug resistance. The ideal antifungal drug should have a broad spectrum, low toxicity, activity in both oral and parenteral administrations, adequate pharmacokinetics, and low cost [4]. However, given the absence of this ideal drug and taking into account the large number of variables that can affect its activity both in vitro and in vivo, it is necessary to continue the search for new antifungal agents.

Actinomycete species belonging to the genus *Streptomyces* are recognized as some of the most valuable sources of secondary metabolites, covering a wide range of structural diversity and biological properties. These include, among others, antifungal, antibacterial, antiviral, antitumoral, anti-hypertensive, and immunosuppressive properties. These secondary metabolites provide unlimited opportunities for discovering novel natural antifungal drugs, because of the matchless availability of chemical diversity [5]. Non-ribosomal peptide synthetases (NRPSs) and polyketide synthases (PKSs) are involved in the biosynthesis of a large number of important antimicrobials produced by actinomycetes, including antibacterial drugs such as tetracycline, erythromycin, and kanamycin, as well as antifungal agents such as candicidin and nystatin, among others [5,6]. The incidence and presence of genes that code these enzymes are used as an alternative to evaluate their biosynthetic potential [7]. *Streptomyces* strains are highly abundant in soil, where the number and type of strains present in a particular soil are greatly influenced by environmental conditions such as temperature, pH, amount of organic matter, aeration etc. [8,9].

Mexico is one of the countries with the greatest biodiversity world-wide, represented by a wide variety of ecosystems that influence variations in the type of soil, thereby providing a very complex habitat for the microbes residing therein [10,11]. In particular, the tropical soils are scarcely explored ecological niches which can be used as a priceless reservoir in the search for natural products from actinomycetes. In this regard, only two previous studies have been reported, which included *Streptomyces* strains obtained from soil collected in the Los Petenes reserve biosphere (state of Campeche) and actinobacteria isolated from cenote waters (state of Yucatan) with activity against *Bacillus subtilis, Escherichia coli,* fungal phytopathogens, and the yeast *C. albicans* [12,13]. In recent studies, actinobacterial strains from tropical acidic soils of the state of Tabasco, in Mexico, were isolated, identified as *Streptomyces* according to their morphological and biochemical characteristics, and screened against several bacterial pathogens [14]. As a continuation of this work, the aim of the present study was to carry out the molecular characterization and evaluation of the antifungal activity against *C. albicans* ATCC (American type culture collection) 10231 of four selected *Streptomyces* strains isolated from tropical acid soil in Tabasco, Mexico. The selected strains were identified using the analysis of 16S ribosomal RNA (rRNA) sequencing; the biosynthetic gene clusters associated with PKS-I, PKS-II, and NRPS were detected, and the presence of antifungal compounds in active extracts of these strains was analyzed using liquid chromatography-ultraviolet-high resolution mass spectrometry (LC-UV-HRMS).

## 2. Materials and Methods 

### 2.1. Streptomyces Strains

The actinobacterial strains were provided by the Autonomous University of Tabasco. These strains were isolated from tropical soils in the state of Tabasco, Mexico. Strain S2A-04 was from Florida (pH 4.2), strain S6A-03 was from Cocona (pH 5.05), and strains S3A-05 and S3A-09 were from Puyacatengo (pH 5.89). The strains were isolated using Internacional Streptomyces Project 2 (ISP2) medium, with incubation temperature and time of 25 °C and 7 days, respectively. The strains were morphologically characterized by gram staining and their corresponding biochemical tests [14].

### 2.2. Culture of the Streptomyces Strains

Cultivation of all of the *Streptomyces* strains first involved the generation of a seed culture. The strains were grown in Internacional Streptomyces Project 2 (ISP2) liquid medium (10 mL), adjusted to pH 7.0, and incubated at 28 °C ± 1 °C with shaking at 125 rpm. After 5 days, mycelia were collected by centrifugation, frozen at −80 °C, and macerated to a powder consistency for DNA extraction. After 7 days of incubation, this seed culture (10 mL) was used to inoculate 90 mL of ISP2 liquid medium (10 %, v/v), and this was incubated under the conditions described above for 15 days. After this period, the culture broth was centrifuged at 3220 × g/15 min at 4 °C to precipitate the cells and the supernatant (SN) was obtained by decantation. Each SN (1×) was reduced to a half of its original volume by lyophilization (Labconco FreeZone 2.5, model 7670520, Houston, TX, USA), obtaining the concentrated SN (2×) which was tested in the initial antifungal bioassay [15]. 

### 2.3. Genomic DNA Extraction, Amplification and Sequencing of 16S rRNA

The DNA from macerated mycelia was extracted according to the method of Tapia-Tussell et al. [16]. Each reaction mixture (50 μL final volume) consisted of 100 ng DNA template, 1× PCR buffer (10× 200 mM Tris-HCl, 500 mM KCl, pH 8.4; Invitrogen), 0.2 mM of dNTP mixture, 0.5 μM of each primer (Invitrogen), 1.5 mM MgCl_2_, and 1.5 U of Taq DNA polymerase (Invitrogen). PCR amplification of the 16S rRNA gene was conducted using the general primers 8F 5’-AGA GTT TGA TCC TGG CTC AG-3´ and 1492R 5-´GGT TAC CTT GTT ACG ACT T-3´. DNA amplification was performed in a C100 touch Thermal Cycler (Bio-Rad, Foster city, CA, USA). The reaction conditions were as follows: initial denaturation at 95 °C for 5 min, 30 cycles of 95 °C for 45 s, 55 °C for 45 s, and 72 °C for 1.5 min, with a final extension of 8 min at 72 °C. Amplified DNA fragments were separated by electrophoresis in 1.5% (w/w) agarose gels (Invitrogen) in 1× Tris-Borate-EDTA (TBE) buffer at 100 V for 40 min, and stained with ethidium bromide. DNA banding patterns were visualized, and images were acquired with a GelDoc EZ imager (Bio-Rad, Hercules, CA, USA) [17]. The PCR products were directly sequenced using the same primers by Macrogen (Seoul, Korea). The 16S rDNA sequences were compared to the sequences within the Ribosomal Database Project (RDP) using the Sequence Match algorithm. The genetic relation between the strains was evaluated based on standard genetic distances, using the software Molecular evolutionary genetics analysis (Mega) 5.1 (Tamura, PL, USA) [18]. Results were used to build a dendrogram with the unweighted pair group method with arithmetic mean (UPGMA), with 1000 bootstrap repetitions. 

### 2.4. Detection of PKS-I, PKS-II, and NRPS Gene Clusters

Polyketide synthase (PKS) type I gene fragments were amplified using degenerate primers: K1F 5′-TSA AGT CSA ACA TCC GBCA-3′ and M6R 5′-CGC AGG TTS CSG TAC CAG TA-3′. Non-ribosomal peptide synthetase (NRPS) gene fragments were amplified using degenerate primers: A3F 5′-GCS TAC SYS ATS TAC ACS TCS GG-3′ and A7R 5′-SAS GTC VCC SGT SGC GTA S-3′ [19]. Gene PKS-II was amplified using the degenerate primer: KSα 5′-TSG CST GCT TGG AYG CSA TC-3′ and KSβ 5′-TGG AAN CCG CCG AAB CCG CT-3′. The amplification was performed in a C100 touch Thermal Cycler (Bio-Rad, CA, USA). Each reaction mixture (25 μL final volume) consisted of 400 ng DNA template, 1× PCR buffer (10× 200 mM Tris-HCl, 500 mM KCl, pH 8.4; Invitrogen), 0.2 mM of dNTP mixture (Invitrogen), 0.4 μM of each primer, 2.5 mM MgCl_2_, 1.5 U of Taq DNA polymerase (Invitrogen), and 10 % dimethyl sulfoxide (DMSO). The PCR amplification program consisted of an initial denaturation at 95 °C for 5 min, followed by 35 cycles of 95 °C for 30 s, an annealing step of 2 min at 55 °C with K1F/M6R, at 58 °C with KSα/KSβ, and at 59 °C with A3F/A7R, with an extension of 4 min at 72 °C, and a final extension at 72 °C for 10 min [19,20]. All amplification products were visualized on 1.5 % (w/v) agarose gels prepared in 1× Tris-Borate-EDTA (TBE) buffer (Invitrogen) and stained with ethidium bromide. DNA banding was visualized, and images were acquired with a GelDoc EZ imager (Biorad, CA, USA).

The bands in ranges of 1200–1400, 800–900, and 700 bp were classified as products of the PKS-I, PKS-II, and NRPS genes, respectively.

### 2.5. Antifungal Bioassay of Supernatants

#### 2.5.1. Microbroth Bioassay of Supernatants of the Actinobacterial Cultures

Microbroth assays were conducted in sterile 96-well plates. The concentrated SNs (2×) were sterilized using 0.22-μm pore size filters (Millipore) and tested against *C. albicans* ATCC 10231. The yeast was grown on Sabouraud’s agar at 35 °C for 24 h and the inoculum was prepared in sterile saline solution (0.85%) and adjusted to match the turbidity of 0.5 McFarland standard at 530 nm (Spectrophotometer SP-830 plus, Nangang, Taipei, Taiwan) comprising 10^6^ colony-forming unit (CFU)/mL^−1^, subsequently diluted at 1:1000 dilution with Sabouraud’s broth. A volume of 100 μL of each SN (2×) was deposited in each microwell, and 100 μL of the dilution of *C. albicans* was added. The final concentration of the SN was 1×, and that of the inoculum was 1–2.5 × 10^3^ CFU/mL. Miconazole (10 μg/mL) and the ISP2 medium were used as the positive and negative control, respectively. Four repetitions were made for each sample of the *Streptomyces* culture supernatants and controls. The microplates were incubated at 35 ± 2 °C for 24 h. After incubation, (1) with a metal replicator, a sample from each of the microwells was transferred onto Sabouraud’s agar plates free of the extracts (replication agar plates), and (2) immediately, 50 μL of 2,3,5-triphenyl tetrazolium chloride (TTC) 1% was added to each microwell. Both cultures, the replication agar plates and microwell plates with TTC 1%, were incubated under the same, aforementioned conditions. Viability data were recorded at 48 h; the absence of fungal growth on the replication plates and the absence of pink or red coloring in the microwells with TTC 1% were interpreted as fungicidal action [21,22].

#### 2.5.2. Minimum Inhibitory Concentration of Lyophilized Supernatants

Minimum inhibitory concentration (MIC) values were determined according to the M27-A3 methodology for microdilution by the Clinical Laboratory Standards Institute (CLSI), with slight modifications. The supernatants of the strains of *Streptomyces* that demonstrated antifungal effect were fully lyophilized in a freeze-dryer (Labconco FreeZone 2.5, model 7670520, Houston, Texas, USA). The lyophilized SNs were dissolved in distilled water (20 mg/mL) and sterilized through 0.22-μm millipore filters (Millipore). These samples were tested at final concentrations of 10, 5, 2.5, and 1.25 mg/mL. The final concentration of inoculum of *C. albicans* was 1–2.5 × 10^3^ CFU/mL. The positive and negative controls were the same as those mentioned above. After incubation at 35 °C for 24 h, the MIC was determined as the lowest concentration which yielded no visible microbial growth in the wells with TTC 1% and on the replicate Sabouraud’s agar plate [21,22].

### 2.6. Antifungal Assay of organic Extracts

#### 2.6.1. Ethanol Extraction of Lyophilized Supernatant from Strains

The lyophilized SNs of *Streptomyces manipurensis* S3A-05 and S3A-09 and *Streptomyces parvisporogenes* (647, 380 mg, respectively) with demonstrated antifungal effect were extracted with ethanol (three times, w/v) at room temperature. The solvents were removed in a rotary evaporator (Ika RV 10 Control, Staufen, Germany) at 40 °C under reduced pressure until dryness. In this way, the ethanol extract (a) and an insoluble fraction corresponding to the residual solid (b) were obtained.

#### 2.6.2. Minimum Inhibitory Concentration of Fractions from Organic Extraction

The organic extracts (a) were dissolved in DMSO at a concentration of 40 mg/mL. The residual solids (b) were treated as lyophilized SN as mentioned above at the same concentrations. The MIC value of the active extracts was determined based on the microdilution method [22], 10 μL (0.4 mg) of each extract were tested using a 2-fold serial dilution method, with final concentrations of 1, 0.5, 0.25, 0.125, 0.062, and 0.031 mg/mL. The final concentration of DMSO did not exceed 0.5 %.

### 2.7. LC-HRMS Analysis

The organic extracts of the three active *Streptomyces* strains were filtered by RP-18C (40–60 μm, Merck) and analyzed by liquid chromatography-mass spectrometry (LC-MS) on an Agilent 1260 RR chromatograph (Santa Clara, CA, USA). A Zorbax SB-C8 column (2.1 × 30 mm), maintained at 40 °C with a flow rate of 300 μL/min was used. Solvent A consisted of 10% acetonitrile and 90% water with 0.01% trifluoroacetic acid and 1.3 mM ammonium formate, while solvent B was 90% acetronitrile and 10% water with 0.01% trifluoroacetic acid and 1.3 mM ammonium formate. The gradient started at 10% B and reached 100% B in 6 minutes; it was then kept at 100% B for 2 minutes and returned to 10% B for 2 minutes to initialize the system. Full diode array UV scans from 100 to 900 nm were collected in 4-nm steps at 0.25 s/scan. Mass spectrometry acquisition was performed on a Bruker maXis HR-QTOF mass spectrometer (Bruker Daltonics GmbH, Bremen, Germany) coupled to the previously described LC system. Ionization of the eluting solvent was obtained using the standard maxis ESI source adjusted to a drying gas flow of 11 l/min at 200 °C and a nebulizer pressure of 40 psig. The capillary voltage was set to 4000 V. High Resolution Mass Spectra (HRMS) were collected from 150 *m/z* to 2000 *m/z* in positive mode. The chemical profiles were compared to internal proprietary databases of more than 900 known active molecules. Database searching was carried out in MEDINA proprietary database of microbial metabolites and the Chapman & Hall Dictionary of Natural Products v25.1 (CRC Press, Boca Raton, FL, USA) [23,24]

## 3. Results

### 3.1. Molecular Identification and Screening of Antifungal Activity of Streptomyces Strains

**Figure 1:**
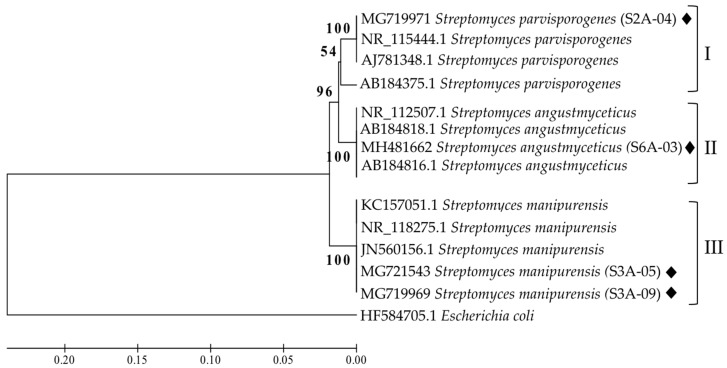
Genetic distance tree grouped by the unweighted pair group method with arithmetic mean (UPGMA) using the 16S ribosomal RNA gene sequence (Ribosomal Date Base Project), showing the genetic relations between the strains in this study (black rhombuses). *Escherichia coli* (HF584705.1) was employed as an outgroup.

The sequencing result of 16S rRNA gene from the four strains showed a similarity level of 100% to other sequences from species belonging to the genus *Streptomyces*. The products of the amplification of the 16S rRNA gene, of approximately 1200 bp of the four strains of *Streptomyces,* were sequenced and blasted against the National Center for Biotechnology Information (NCBI) Gen Bank database. The results are shown in Table 1. The strains S2A-04 and S6A-03 were identified as *Streptomyces parvisporogenes* and *Streptomyces angustmyceticus*, respectively, while both S3A-05 and S3A-09 were identified as *Streptomyces manipurensis*. To corroborate this identification, a phylogenetic tree based on gene distance using the UPGMA method was built (Figure 1). The four strains were separated into three clades. In clade I, the strain S2A-04 grouped with the species *S. parvisporogenes*, in clade II, S6A-03 grouped with *S. angustmyceticus*, and in clade III the strains S3A-05 and S3A-09 grouped with *S. manipurensis* species.

The results of the evaluation performed in the microdilution broth assay against *C. albicans* indicated that all supernatants except that of *S. angustmyceticus* showed activity. The *S. manipurensis* S3A-05 and S3A-09 strains displayed both MIC values of 2.5 mg/mL, while *S. parvisporogenes* S2A-04 exhibited a MIC value of 5 mg/mL (Table 2). All active strains exhibited a fungicidal effect.

The ethanol extracts from the lyophilized SNs of *S. parvisporogenes* S2A-04 and *S. manipurensis* S3A-05 and S3A-09 cultivated in ISP2 were subsequently tested against *C. albicans*. Only the ethanol extract from *S. parvisporogenes* (S2A-04) inhibited the growth of the pathogenic yeast, with an MIC value of 0.5 mg/mL showing fungicidal effect. The ethanol extracts of both *S. manipurensis* strains showed no inhibitory activity on *C. albicans*. The solid residual fractions obtained from the three strains were not active either (Table 2).

### 3.2. Detection of Biosynthetic Genes of Active Strains

**Figure 2:**
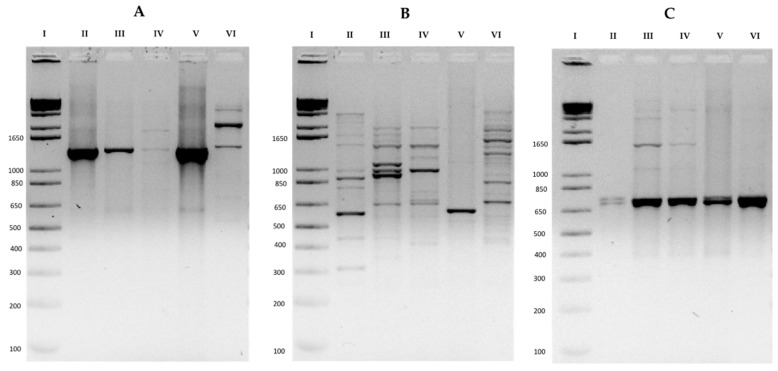
Agarose gel electrophoresis of the biosynthetic gene amplification products. (**A**) 1200–1400 bp fragments of polyketide synthase I (PKS-I) genes, (**B**) 800 to 900 bp fragments of type II polyketide synthase (PKS-II) genes, and (**C**) 700 bp fragments of non-ribosomal peptide synthase (NRPS) genes. Lane, I: 1 kb molecular weight marker; lane II: *Streptomyces parvisporogenes* (S2A-04); lane III: *Streptomyces manipurensis* (S3A-05); lane IV: *Streptomyces manipurensis* (S3A-09); lane V: non-active strain *Streptomyces angustumyceticus* S6A-03; and lane VI: *Streptomyces coelicolor* (control + PKS-I and NRPS).

The presence of PKS-I, PKS-II, and NRPS biosynthetic genes in the *Streptomyces* strains under study was analyzed. PKS-I and NRPS genes were present in all four of the strains (Figure 2A,C). Additionally, the strains *S. parvisporogenes* (S2A-04) and *S. manipurensis* (S3A-05) presented a band (Figure 2B) belonging to expected amplicons (900 bp) for PKS-II type genes (Table 3). According to the results, differences in the biosynthetic potential of *S. manipurensis* S3A-05 and S3A-09 strains were detected.

### 3.3. LC-UV-HRMS Analysis

**Figure 3:**
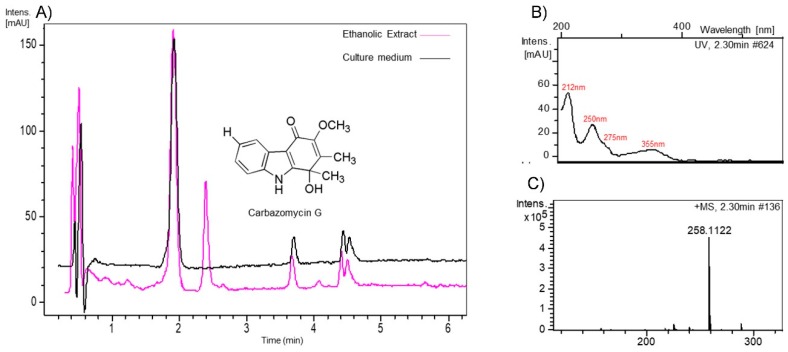
(**A**) LC-UV chromatogram (210 nm) of the ethanolic extract of the *S. parvisporogenes* (S2A-04) strain vs. an extract of the non-fermented culture medium, (**B**) Ultraviolet spectra and (**C**) HRMS spectra of the major peak at 2.30 min and structure of carbazomycin G.

The ethanolic crude extracts of the three bioactive *Streptomyces* strains were analyzed by LC-UV-HRMS. The HRMS of a major peak eluting at a retention time of 2.30 min in the extract of *S. parvisporogenes* displayed a protonated ion at *m/z* 258.1122, in accordance with a molecular formula of C_15_H_15_NO_3_ (calc. for C_15_H_16_NO_3_^+^, 258.1130), and its UV spectrum presented maxima at 212, 250, 275, and 355 nm (Figure 3). Dereplication of that molecular formula and UV features using the Chapman & Hall Dictionary of Natural Products identified carbazomycin G as the major component of the extract. The LC-UV-HRMS analysis of both *S. manipuresis* strains showed no significant differences with respect to that of an extract of the culture medium (blank).

## 4. Discussion

The analysis of the 16S rRNA gene sequences confirmed that the four tropical strains studied herein belong to three different species of the *Streptomyces* genus, *S. angustmyceticus*, *S. manipurensis*, and *S*. *parvisporogenes.* These isolations represent the first contribution to the description of actinobacteria isolated from the state of Tabasco, enriching the knowledge of the bacterial biodiversity present in the southeast of Mexico. The *Streptomyces* strains analyzed belong to species not commonly isolated. In particular, this is the third report of *S. manipurensis* species [25,26]. Our isolates were obtained from acidic soils; these data are in accordance with previous results indicating that the most frequently found acidotolerant actinomycetes in acid soils are representative of the *Streptomyces* genus. This fact is most likely related to their significant predominance in almost all the soils [27,28,29]. Our *S. manipurensis* S3A-05 and S3A-09 strains come from tropical slightly acidic soils, while the only two previously isolated were obtained from a tropical basic (pH 9.26) soil of limestone quarry at Hundung (Manipur India) [28,30] and from calcareous soils in Egypt [31]. The other two strains, *S. parvisporogenes* S2A-04 and *S. angustmyceticus* S6A-03, come from soils with higher acidity. In 2011, Kulkarni-Almeida [32] isolated *S. parvisporogenes* PM0324667 from a neutral to alkaline sandy soil (Rajasthan, India) [33]. Pithakkit [34] reported the isolation of *S. angustmyceticus* NR8-2 from slightly acidic soils from palm tree plantations in the south of Thailand [35]. The *Streptomyces* strains studied in this research, according to the pH of the soil where they were isolated, could be considered as moderately acidophilic [36]. Other actinomycetes living in Mexican tropical soils have scarcely been investigated.

The SNs that showed the most potent antifungal effect against *C. albicans* belong to the *S. manipurensis* S3A-05 and S3A-09 strains. Previous studies of *S. manipurensis* MRLB 201 and isolate H21, cultured in starch–nitrate–casein broth and soybean broth, respectively, reported a strong inhibitory effect on several pathogenic bacteria and lower activity against *C. albicans* by the agar well diffusion method [26,37]. The ethanolic extract and the residual solid obtained from both lyophilized SNs of *S. manipurensis* showed no antifungal activity. This fact might indicate the existence of a synergistic effect among metabolites of both fractions which is lost when they are separated during the ethanolic extraction [38]. Alternatively, the antifungal activity might be related to the presence of proteins as in *Nocardiopsis dassonvillei* [39] or enzymes such as chitinases and glucanases [40,41], capable of degrading the cell wall of the fungi but labile to hydrophilic solvents [42]. 

The PKS-I and NRPS biosynthetic genes detected in all four of the strains studied have also been frequently found in other species *Streptomyces* genus [43,44,45]. The presence of these genes in our strains indicates their potential to biosynthesize macrolide, polyether, and non-ribosomal peptide-like molecules, among others [46,47]. The PKS-II genes detected in the strains *S. manipurensis* S3A-05 and *S. parvisporogenes* provide information regarding their capability to biosynthesize aromatic polyketides [48]. Several studies associate the presence of the PKS-II gene in actinomycete strains with antifungal activity [44,49]. Interestingly, different biosynthetic gene cluster profiles were detected in both *S. manipurensis* strains, even when they have same origin, suggesting differences in their chemical and fingerprint profile. However, to date no metabolites have been isolated from *S. manipurensis* and our initial assessment of their chemical composition on organic extract by LC-UV-HRMS revealed no significant differences between the composition of the actinomycete extracts and the culture medium. To fully understand the biosynthetic capacity of our strains, perhaps new cultivation strategies should be attempted. Among them, the most straightforward will be the use of one strain-many compounds (OSMAC) approaches [50] or microbial co-cultivation experiments [51], which for sure will elicit the production of new natural products that might have applicability in the fight against fungal or other microbial infections.

On the other hand, the initial low anti-*Candida* activity observed for *S. parvisporogenes* S2A-04 was significantly increased after organic extraction of the lyophylized supernatant, leading to a final MIC value of 0.5 mg/mL. Similar results have been reported with ethyl acetate extracts from *Streptomyces* sp. ERINLG-51 [52]. In this case, our results indicated that the existence of ethanol soluble compounds in the extract is responsible for the antifungal activity observed. Based on its HRMS and UV spectra, a metabolite tentatively identified as carbazomycin G was detected as one of the major components of our *S. parvisporogenes* S3A-04 ethanolic extract. This is an aromatic alkaloid related to the presence of PKS-II genes in the strain. In contrast, various secondary metabolites have been identified from other *S. parvisporogenes* strains. These include an antifungal polyene named PA 616 by Chas [53], acetyl, butyl, and propionyl pepstatins with inhibitory activity of pepsin, cathepsin D, and renin [54]. Additionally, an aromatic metabolite, NFAT-33, with antidiabetic properties was isolated from *Streptomyces* sp. PM0324667, a striking strain related to *S. parvisporogenes,* according to their DNA sequencences [32]. Carbazomycin G was previously reported from *Streptoverticillium ehimense* together with carbazomycins A–F and H. It is an alkaloid with a moderate antifungal activity against species of *Trichophyton* [55,56,57]. The structure of carbazomycins possesses a carbazol core originating from a tryptophan unit, coupled to a pyruvate and an acetate unit, pointing to a possible relation with the PKS genes existing in the strain [58,59]. The total synthesis of this compound has been reported by several authors [60,61,62]. It will be necessary to isolate the active compound in order to confirm its activity and its structure in the ethanolic extract from *S. parvisporogenes* S2A-04. 

## 5. Conclusions 

A complete characterization of four Actinomycete strains isolated from Mexican soils was performed. This investigation reveals that the organic extract of one of them, *S. parvisporogenes* (S3A-04), possesses promissory activity against *C. albicans*. It presented the three biosynthetic gene cluster types (NRPS, PKS I, and PKS II), and the metabolite carbazomicin G was identified as the major component of its extract, making this strain attractive for future biotechnological applications. Two new registers of non-common actinobacterial *S. manipurensis* strains are also reported in this work. Extracts of these two strains lost their initially detected antifungal activity after lyophilization of the SNs and extraction with ethanol. On the other hand, the presence of PKS and NRPS genes in all four of the strains studied indicates the existence in their genomes of a biosynthetic potential that perhaps was not fully expressed under the single culture conditions tested in our study. Finally, our study also confirms that actinomycetes from tropical Mexican soils constitute an important source in the search for alternative anti-*Candida* products.

## Figures and Tables

**Table 1:** 16S ribosomal RNA gene taxonomic affiliation of *Streptomyces* strains isolated from tropical acidic soils in the state of Tabasco, Mexico.

Strain	Accesion Number GenBANK	Closest Relative by BLAST	% SEQUENCE Similarity
S2A-04	MG719971	*Streptomyces parvisporogenes* (AJ7813481)	100
S3A-05	MG721543	*Streptomyces manipurensis* (JN560156.1)	100
S3A-09	MG719969	*Streptomyces manipurensis* (NR118275.1)	100
S6A-03	MH481662	*Streptomyces angustmyceticus* (AB184816)	100

**Table 2:** Minimum inhibitory concentration (MIC) of supernatants and fractions of *Streptomyces* sp. collected in tropical acidic soils of the state of Tabasco, Mexico.

Strains	pH Soil	Collection Site		MIC (mg/mL)		
			SN (1×) Activity	Lyophilized SN	Ethanol Extract	Residual Solid
*Streptomyces parvisporogenes* S2A-04	4.2	Florida	**+**	5	0.5	>1.0
*Streptomyces manipurensis* S3A-05	5.89	Puyacatengo	**+**	2.5	>1.0	>1.0
*Streptomyces manipurensis* S3A-09	5.89	Puyacatengo	**+**	2.5	>1.0	>1.0
*Streptomyces angustmyceticus* S6A-03	5.05	Cocona	**−**	>10	NE	NE

+: Active, −: Not active. NE: Not evaluated. SN: Supernatant (1×): original concentration tested bioassay.

**Table 3:** Detection of biosynthetic genes in active *Streptomyces* strains.

Strains	Gene Type		
	PKS-I	PKS-II	NRPS
*S. parvisporogenes* (S2A-04)	+	+	+
*S. manipurensis* (S3A-05)	+	+	+
*S. manipurensis* (S3A-09)	+	−	+
*S. angustmyceticus* (S6A-03)	+	−	+

+: Gene present; −: Gene not present.
